# The Effect of Correcting Neuromyths on Students’ and Teachers’ Later Reasoning

**DOI:** 10.3390/jintelligence12100098

**Published:** 2024-10-01

**Authors:** Marcus Per Gustaf Lithander, Lisa Geraci, Meltem Karaca, Renee Hunsberger

**Affiliations:** 1 Division of Digital Learning, KTH Royal Institute of Technology, 10044 Stockholm, Sweden; 2 Department of Psychology, University of Massachusetts Lowell, 850 Broadway Street, Lowell, MA 01854, USA; lisa_geraci@uml.edu (L.G.); renee_hunsberger@student.uml.edu (R.H.); 3 Department of Psychology, Assumption University, 500 Salisbury Street, Worcester, MA 01609, USA; m.karaca@assumption.edu

**Keywords:** continued influence effect, neuromyths, misconceptions, refutation text, reasoning, explanations

## Abstract

Students and educators sometimes hold beliefs about intelligence and learning that lack scientific support, often called neuromyths. Neuromyths can be problematic, so it is important to find methods to correct them. Previous findings demonstrate that textual refutations are effective for correcting neuromyths. However, even after correction, erroneous information may continue to influence reasoning. In three experiments, we investigated whether feedback could be used to update students’ and educators’ beliefs and influence their reasoning about neuromyths. Across all experiments, the results showed that both students and educators held erroneous beliefs about learning and memory that could be updated after receiving feedback. Feedback also increased students’, but not teachers’, reasoning accuracy. The results demonstrate that feedback can be used to update beliefs in neuromyths, but these beliefs may influence reasoning even after correction.

Research suggests that many students and educators hold unsupported beliefs about the brain and cognition, particularly those focused on intelligence and learning, often called neuromyths (Blanchette Sarrasin et al. 2019; Dekker et al. 2012; Deligiannidi and Howard-Jones 2015; Dündar and Gündüz 2016; Ferrero et al. 2016, 2020; Gleichgerrcht et al. 2015; Howard-Jones 2014; Khramova et al. 2023; Karakus et al. 2015; Lithander et al. 2021; Macdonald et al. 2017; Papadatou-Pastou et al. 2017; Pei et al. 2015; for a recent review see Torrijos-Muelas et al. 2021). For example, many people believe that learners should be classified as either “left-brained” or “right-brained” and that this characteristic predicts learning (Macdonald et al. 2017) despite there being no evidence for such an influence on learning (Corballis 2014; Nielsen et al. 2013; van Dijk and Lane 2020). Beliefs in these neuromyths can be problematic, as they can influence teaching and learning practices (Blanchette Sarrasin et al. 2019; Ferrero et al. 2020). For example, the myth that students learn best when information is presented in their preferred learning style may lead students to withdraw from learning activities that they deem “unfit” for them or to invest in costly learning-style assessments (Newton and Salvi 2020; Pashler et al. 2008; Willingham et al. 2015). More broadly, holding false beliefs about intelligence and learning can lead teachers and students to spend valuable time and resources on ineffective strategies instead of using effective strategies (Dunlosky et al. 2013). One consequence of this potential misuse of resources is that students may experience poor academic outcomes (Dunlosky et al. 2013; Morehead et al. 2016).

To optimize educational resources and make room for evidence-based strategies, it is important to refute neuromyths (Krammer et al. 2021; Rousseau 2021). Past research suggests that textual refutations can be used to update erroneous beliefs in general (Chan et al. 2017; Rich et al. 2017; Swire and Ecker 2018). Various types of refutation texts have been effectively used to correct erroneous beliefs about educational misconceptions, including those simply stating that the information is incorrect, to more complex refutations that include explanatory and personalized feedback (Lithander et al. 2021; Ferrero et al. 2020; Dersch et al. 2022). This is performed by first presenting the misconception (e.g., “Bulls are enraged by the color red”), followed by a refutation (e.g., “Many people think that the color red enrages bulls, but this notion is false; Rich et al. 2017; Van Loon et al. 2015). Presenting both false and new (correct) information together may help people revise and update their knowledge (Kowalski and Taylor 2017; Lewandowsky et al. 2012; Van Loon et al. 2015). Refutations have been used to correct students’ beliefs in neuromyths in particular. For example, Lithander et al. (2021) presented participants with a list of neuromyths (e.g., “We only use 10% of our brains”), followed by various types of refutations (e.g., “This statement is false. Although it is commonly believed that we only use 10% of the brain, this notion is false”). The results showed that all types of refutations (those with and without explanations) were effective in correcting students’ erroneous beliefs in neuromyths. Further, this corrective effect held for one week and one month (but see also Swire-Thompson et al. 2023; Swire et al. 2017; Paynter et al. 2019).

Despite these promising findings, other research shows that beliefs in neuromyths can persist even after a correction. For example, Ferrero et al. (2020) used refutations to correct teachers’ beliefs in neuromyths. The results revealed that teachers’ initial misconceptions could be corrected in the short term but returned after a one-month delay. Further, teachers reported that they intended to transfer these ideas (the misconceptions) to the classroom. Therefore, it remains unclear how effective refutations are over time and if the effectiveness of refutations differs between students and teachers. There are several differences between the studies by Lithander et al. (2021) and the studies by Ferrero et al. (2020) that could offer insights into when and for whom refutations will be most effective. For example, teachers may be more susceptible to returning to prior beliefs than are students due to greater exposure to true and false pedagogical information (Walter and Tukachinsky 2020).

Not only might it be difficult for some people to update their knowledge, but even when people appear to update their knowledge (they correctly identify the information as false), they may continue to rely on earlier knowledge when reasoning about various situations (see Ecker et al. 2011; Guillory and Geraci 2010). For example, teachers may be able to correctly identify that learning styles do not promote student learning but still intend to use these various presentation modes to match students’ potential learning styles (see Newton and Miah 2017). In other domains, research suggests that people may continue to rely on false information for reasoning even after a correction (the continued influence effect; Ecker and Antonio 2021; Guillory and Geraci 2013, 2016; Johnson and Seifert 1994; Lewandowsky et al. 2012).

The current studies examined whether corrective feedback is effective for updating reported beliefs in neuromyths and reasoning based on neuromyths. We hypothesized that though people may explicitly change their response to indicate that a neuromyth is incorrect following a correction, they may continue to believe in neuromyths and use them to reason about effective learning strategies. Therefore, we designed a task to measure whether people are using lingering beliefs in neuromyths to reason about learning in real-world contexts. Participants read scenarios describing a learner’s study behaviors that were either based on beliefs in neuromyths or on supported evidence-based research. Participants were asked to indicate whether they agreed with the person’s reasoning illustrated in each scenario. This paradigm allowed us to investigate whether corrective feedback could be effective for updating not only beliefs in statements, as shown before but also reasoning about real-world learning scenarios. Previous research indicates that using scenarios can enhance the assessment of participants’ knowledge (Tovazzi et al. 2020). Therefore, we used a method that incorporates scenario evaluations. We investigated students’ and teachers’ use of corrective feedback to update their beliefs and how these corrected beliefs may influence reasoning. Ferrero et al. (2020) found that refutations can be used to update educators’ reported beliefs in neuromyths. However, refutations did not change teachers’ intention to implement the neuromyths in their teaching practices. Thus, we examined whether feedback can be used to influence students’ and teachers’ *reasoning* about learning practices. We hypothesized that it might be particularly challenging to affect teachers’ reasoning (and not just reported beliefs) following a refutation because of engrained beliefs about learning resulting from extensive training and experience (Walter and Tukachinsky 2020). Students (Experiments 1 and 2) and teachers (Experiment 3) read true and false statements (neuromyths) about intelligence and learning and indicated the veracity of the statements before receiving either corrective feedback or no feedback. Later, they were asked about the veracity of the initial statements, and novel to this study, they were asked to indicate their level of agreement with the reasoning described in various learning scenarios.

## 1. Experiment 1

In Experiment 1, we examined the effects of refutative feedback on students’ reported beliefs (agreement with the statements) and reasoning (agreement with the scenarios). To assess beliefs, participants were asked to indicate whether various statements about learning were true or false before and after receiving feedback, following the paradigms used by Lithander et al. (2021) and Ferrero et al. (2020). To assess reasoning, participants read various learning scenarios and indicated if they agreed with the reasoning outlined in the scenarios before and after receiving feedback or no feedback. For example, for the neuromyth that individuals learn better when they receive information in their preferred learning style, the scenario described a student who endorsed being a kinesthetic learner and therefore decided to be physically active while studying. Participants were asked to indicate their agreement with the person’s reasoning. Two types of feedback were used in Experiment 1, feedback-only and feedback with explanatory text. The hypothesis for Experiment 1 was that providing students with feedback, compared to no feedback, would reduce participants’ reported beliefs in neuromyths, consistent with previous research (see Lithander et al. 2021). Also, we predicted that providing students with feedback, compared to no feedback, would not effectively reduce participants’ reliance on the neuromyths during reasoning (scenarios). Finally, we examined whether providing feedback that included explanatory text or no explanatory text would be more effective for updating knowledge and reasoning, as previous findings show different results regarding the effect of type of feedback (e.g., Rich et al. 2017; Ferrero et al. 2020; Lithander et al. 2021).

### 1.1. Method

Materials and data for all experiments are available on the Open Science Framework: https://osf.io/s85e4/?view_only=861260760bdc412a8d48e8eb8f5083b1. Accessed on 21 April 2023.

#### 1.1.1. Participants

Based on previous work on correcting neuromyths using a similar design (Rich et al. 2017), we selected an effect size of *t* = 14.91, (η_p_2 = 0.12), 1 − β = 0.80 power at α = 0.05 to calculate how many participants would be needed to detect a significant difference between each of the feedback conditions. We used the BUCSS package (Anderson and Kelley 2017; Taylor and Muller 1996) in R and a 75% assurance to control for uncertainty and 0.05 rate of publication bias. This power analysis suggested that we needed a sample size of 99 participants (33 participants per condition). To help ensure that we would obtain complete data from at least 99 participants, we recruited additional participants.

One hundred and thirty-three students from a large northeastern university in the United States initiated the study. Participants who did not complete the full study were excluded from the analyses. Consequently, the final data set included 110 participants (59 female; 17–46 years old, *M* = 21.06, *SD* = 4.86). The sample size was based on previous studies using similar methods (Lithander et al. 2021; Rich et al. 2017; Van Loon et al. 2015). Participants who completed both the initial and delayed tests were compensated with course credit. Because we did not have specific predictions about differences across majors, we did not collect this information for specific students who participated in each study. However, we do know the majors for students on SONA participant system for the period when the data were collected: 13.02% biology, 12.86% nursing, 10.95% engineering, 10.79% business, 10.16% exercise science, 5.87% criminal justice, 4.76% computer science, 4.28% liberal arts, 3.33% psychology, 3.17% political science, 3.02% chemistry, 3.02% education, and 2.06% pharmaceutical sciences, with all other majors making up less than <2% of the students on SONA. The University Institutional Review Board approved all experimental procedures, and all participants provided informed consent.

#### 1.1.2. Design and Materials

Experiment 1 used a between-subjects design (no feedback, feedback only, and feedback–explanation). There were two dependent variables of interest: beliefs and reasoning (as measured by participants’ responses to the statements and scenarios). Participants were randomly assigned to either the no-feedback (*n =* 33), feedback-only (*n =* 34), or feedback–explanation (*n =* 43) condition. In the no-feedback condition, participants re-read each statement with no feedback included. Participants in the feedback-only condition read a short text after each statement indicating that the statement was true or false (e.g., “this statement is false”). Participants in the feedback–explanation condition received the same information as those in the feedback-only condition, but for each statement, a longer explanation was added, following the standard format for a refutation.

The materials included 20 true statements (those supported by empirical research) and 20 false statements (those with no or insufficient evidence; *neuromyths*) about learning and the brain. True and false statements were randomly intermixed to avoid participants generalizing their responses across items. These stimuli were taken from Lithander and colleagues, with the exception of four items that were reworded to better translate into real-world scenarios. Scenarios were created based on the 40 true and false statements and modeled from studies investigating the continued influence effect (e.g., Ecker and Antonio 2021; Guillory and Geraci 2010, 2013). Specifically, scenarios were constructed so that participants would need to make inferences about the information they read. For example, for the misconception “*Individuals learn better when they receive information in their preferred learning style* (e.g., *auditory; visual; kinesthetic).*”, participants were presented with the following scenario and asked to indicate their agreement with the person’s reasoning. Participants read the following: “*Juan has identified that he is a kinesthetic learner. Juan feels that kinesthetic learners can benefit from being physically active while studying. Therefore; Juan decides to move around and chew gum while reading his textbook for a class.*” Consistent with methods used in previous research investigating the continued influence effect, participants completed a scale asking “I find the person’s reasoning to be…” using a 7-item Likert scale from 1 (definitely false) to 7 (definitely true) (Guillory and Geraci 2013; Johnson and Seifert 1994; Wilkes and Leatherbarrow 1988).

#### 1.1.3. Procedure

For the initial test, participants were presented with each statement one at a time, with true and false statements randomly intermixed. After reading each statement, participants indicated whether the statement was true or false using a dichotomous selection (see Lithander et al. 2021; Rich et al. 2017; Van Loon et al. 2015 for similar procedures). Next, participants indicated their confidence level in their answers on a scale from 0 (not at all confident) to 100 (absolutely confident). We examined participants’ initial confidence because previous findings suggest that the initial confidence in which people hold erroneous beliefs can influence the effectiveness of the refutative feedback (Ecker et al. 2011; Van Loon et al. 2015). In addition, people may endorse a belief (for example, that a neuromyth is false) but not be confident in this belief (Salovich et al. 2021). Thus, including confidence may give us more insight into participants’ beliefs. Following Lithander et al. (2021), we measured the potential role that the belief had in participants’ lives by asking participants to indicate if they strongly disagree (0) or strongly agree (100) with the following statement: “*This concept has influenced my behavior in daily life”*. We included this measure because previous research suggests that having a personal connection to information can influence how it is assessed and whether the information can be corrected (Lewandowsky et al. 2012; Wells et al. 2009). Because knowing the source of various neuromyths is potentially useful, participants indicated where they remembered hearing about each statement.

After evaluating all 40 statements, participants completed a distractor task requiring them to complete 20 simple mathematical problems; each was displayed for six seconds for two minutes. Next, participants completed the feedback phase of the experiment, in which all 40 statements were presented again in a randomized order. For false statements, the feedback refuted the misconception. For true statements, the feedback affirmed the statement. Feedback was displayed for 25 s, and then, the screen automatically advanced to the next item. After all statements were presented and refuted or not, participants were reminded to return for the second delayed test and dismissed.

Seven days later, participants completed the delayed test. In this session, participants evaluated the same statements as those presented during the initial test and 40 real-world learning scenarios that relied on facts or neuromyths from the initial test. The order of evaluation (statement vs. scenario first) was counterbalanced, and a 2 min distractor task consisting of 20 simple math problems was placed between the blocks. When evaluating the statements, participants again indicated whether they were true or false, following the procedure used in the initial test. When evaluating scenarios, participants completed the statement, “I find the person’s reasoning to be…” with a 7-point Likert scale from 1 (definitely true) to 7 (definitely false) and rated their confidence in this belief as in the initial test. Next, participants completed a brief demographic questionnaire and short versions of trait measurements used in previous research on misconceptions (Bensley et al. 2014; Bensley and Lilienfeld 2017; Swami et al. 2016). The scales used were (Big Five Inventory (BFI-2-S), including six items assessing openness to new experiences (Soto 2019), an 11-item Critical Thinking Disposition Scale (Sosu 2013), and an 18-item Need for Cognition Scale (Cacioppo et al. 1984).

#### 1.1.4. Analytic Plan

Because we were interested in whether refutative feedback was effective for updating beliefs and reasoning of neuromyths, the analyses were focused on false statements only. Research suggests that neuromyths may not align into one overarching construct (Horvath et al. 2018; Macdonald et al. 2017). Therefore, multilevel models were used to account for differences across items (different neuromyths) and individuals (random effects) (Bates et al. 2014; Judd et al. 2017). We aimed to build two models, one that predicted belief after delay and one that predicted reasoning (the scenarios), also measured after delay. For each dependent variable of interest, we built a series of random coefficients and intercepts and slopes as outcome models with statement-level variables (e.g., confidence in belief) standardized using RobustHD. Model selection was based on the likelihood ratio tests and BIC and AIC value comparisons. These models are reported in-text. We note that there is concern about using variables collected after a manipulation as part of a causal estimate (e.g., Montgomery et al. 2018). In the final model, confidence in delayed judgments was the only variable that was collected after the manipulation (refutative feedback). However, the results of the study did not change with the inclusion of this variable, so we retained this variable in the analyses because it improved model fit. The tables with the alternate analyses for each experiment can be found on OSF.

Beliefs (measured with the statements) and reasoning (measured with the scenarios) were measured using different scales, a binary True/False dichotomous scale and a 7-point Likert scale, respectively. Therefore, for the analysis of Experiment 1, we used a logit model to analyze beliefs on the delayed test, whereas reasoning was treated as continuous. We used these two types of scales because previous research examining reasoning and inferences typically uses a Likert scale (Guillory and Geraci 2013; Walter and Tukachinsky 2020), whereas studies investigating beliefs in neuromyths typically use a binary true/false scale (Dekker et al. 2012). However, to anticipate the results of Experiments 2 and 3, the results remain largely the same regardless of the type of scale. When reporting means and standard deviations within tables and figures, Likert scales were transformed to binary scales (which do not affect significance or parameter values).

### 1.2. Results and Discussion

#### 1.2.1. Initial Beliefs

The initial mean scores for beliefs in neuromyths did not differ between conditions, which was expected, as the initial test preceded the feedback manipulation. Table 1 shows the average and standard deviation values for scenarios are reported based on the Likert scales rescored to 0–1.

#### 1.2.2. Effect of the Corrective Feedback on Statement Accuracy

**Figure 1:**
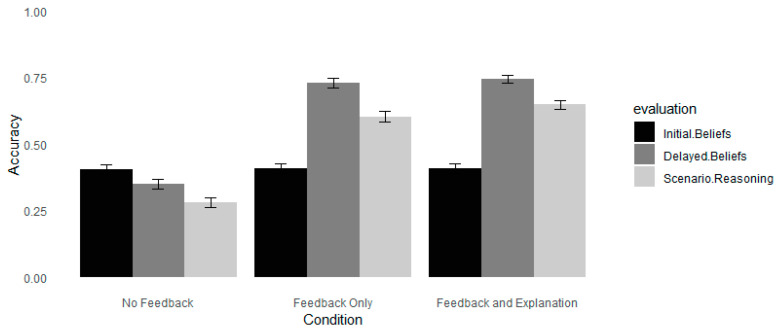
The accuracy of evaluating neuromyths and scenarios in Experiment 1. *Note*. Error bars depict standard deviations. Accuracy was calculated as the average score recorded on the Likert scale (Likert scale 1–5).

Estimates showed that students in the feedback conditions improved their accuracy significantly compared to their accuracy on the initial test (see Table 2). Estimates also suggest that accuracy between the feedback-only and feedback–explanation conditions did not differ, *p* = .46. These results replicate the findings from Lithander et al. (2021), showing that adding explanations did not improve participants’ accuracy in detecting neuromyths. The results also demonstrated that students in the control condition were less accurate in their judgments of neuromyths at the delayed test phase compared to the initial test (see Figure 1). This finding may be due to the fact that participants were re-exposed to the statements during the feedback phase (Dechêne et al. 2010; Fazio et al. 2015).

In addition to a significant effect of the condition, accurate initial test performance increased the probability of accurate delayed test performance (β = 1.27). The results also suggest that for each increase in reported confidence on the delayed test, the more likely participants were to accurately respond to the statements (provide higher Likert scale ratings indicating that the neuromyth was false; β = 0.38). The model also suggested that for each increase in reliance on the neuromyth, students were less likely to update their erroneous beliefs (β = −0.28).

#### 1.2.3. Effect of Corrective Feedback on Reasoning Accuracy

A separate regression model was used to investigate the effect of feedback on participants’ reasoning. In this model, the dependent variable was participants’ ratings of the reasoning outlined in each of the scenarios on a 7-point Likert scale (1 = agree and 7 = disagree; see Table 3). Estimates showed that students who received feedback (both feedback only and feedback–explanation) relative to students in the no-feedback condition, provided higher ratings (were more likely to disagree with the reasoning based on the neuromyths). The initial belief accuracy also significantly predicted the later reasoning accuracy. Accurate beliefs on the initial test were predicted to increase the scenario rating by 0.50 in the delayed test. Similarly, higher confidence in scenario evaluation (on the delayed test) predicted more accurate scenario evaluation accuracy (β = 0.33). Unlike the belief results, reliance on neuromyths in life did not significantly predict accurate judgments of reasoning, *p* = .46.

In sum, the results from Experiment 1 showed that corrective feedback with and without explanations was effective for updating participants’ erroneous beliefs about learning and the brain. Also, both types of corrective feedback aided in the accurate evaluation of reasoning depicted in the scenarios. These findings suggest that feedback is effective for updating both beliefs and reasoning.

## 2. Experiment 2

The goal of Experiment 2 was to replicate the results from Experiment 1 and to determine if the effects of feedback on beliefs and reasoning held over a longer period of time—a month. Previous studies demonstrate that the effect of refutations on beliefs can dissipate after a one-month delay (Ferrero et al. 2020). In addition, previous research shows that intentions to apply educational misconceptions can return to initial levels after a one-month delay (Ferrero et al. 2020). Thus, in Experiment 2, we used a one-month delay between the feedback phase and the final tests of the statements and scenarios.

### 2.1. Method

#### 2.1.1. Participants

Based on the results of Experiment 1 (*F* = 13.65, 0.80 power, α = 0.05), we conducted a new power analysis to estimate the number of participants needed in Experiment 2 to detect a difference between the control and feedback conditions using an assurance of 75% to account for uncertainty. This power analysis suggested that we needed a minimum sample size of 102 participants. As in Experiment 1, we recruited additional participants to ensure that we would obtain the required number of participant data.

Two hundred and one students from a large northeastern university in the United States who had not participated in Experiment 1 were enrolled in the study. Participants who did not finish the full study were excluded from analyses. Consequently, the final data set contained 122 of participants (50 female, 18–58 years old, *M* age = 20.13, *SD* = 4.45). Participants who completed all parts were compensated with course credit. All procedures were approved by the Institutional Review Board, and all participants provided informed consent.

#### 2.1.2. Design and Materials

As in Experiment 1, Experiment 2 used a between-subjects design (no feedback, feedback only, and feedback–explanation). There were two dependent variables of interest: beliefs and reasoning (as measured by participants’ responses to the statements and scenarios) that were both measured after 30 days. The order of the dependent measures (statements versus scenarios) was counterbalanced.

The materials used in Experiment 2 were identical to those used in Experiment 1, with the exception that participants were asked to indicate whether they thought the statements and scenarios were true or false using a 5-point Likert scale. This change was made to align all the scales in the initial test phase. To better assess the extent to which people rely on neuromyths, we also modified the wording of the question about daily influence on one’s life from: *“This concept has influenced my behavior in daily life” to “This concept has influenced how I study”.*

#### 2.1.3. Procedure

The procedure was identical to the procedure used in Experiment 1, with the exception that an additional delay of 30 days was included.

#### 2.1.4. Analytic Plan

As in Experiment 1, we were interested in whether refutative feedback was effective for updating beliefs and influencing reasoning of neuromyths, so the analyses were focused on false statements only. A mixed regression model was used to analyze the effect of corrective feedback on beliefs and how feedback influenced reasoning. Unlike Experiment 1, participants indicated their belief in the statements about learning and the brain by using a scale from 1 (strongly agree) to 5 (strongly disagree). Thus, the dependent variable was analyzed as a continuous variable, with higher ratings indicating that participants disagreed with the neuromyth (they detected it as being false). Analyses for reasoning remained the same, although the scale was changed from a 7-point Likert scale to a 5-point Likert scale to be consistent with the belief rating scale. As in Experiment 1, when reporting means and standard deviations in the tables and figures, Likert scales were transformed to binary scales (which does not affect significance or parameter values).

### 2.2. Results and Discussion

#### 2.2.1. Initial Beliefs

The initial mean scores for beliefs in neuromyths did not differ between conditions, which was expected, as the initial test preceded the feedback manipulation (see Table 4).

#### 2.2.2. Effect of the Corrective Feedback on Statement Accuracy

**Figure 2:**
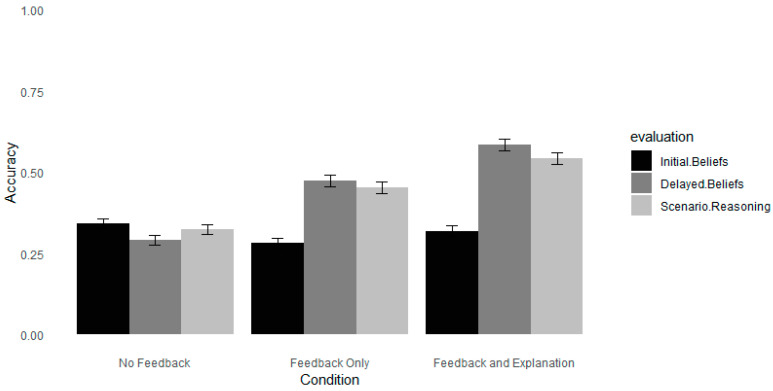
The accuracy of evaluating neuromyths and scenarios in Experiment 2. *Note*. Error bars depict standard deviations. Accuracy was calculated as the average score recorded on the Likert scale (Likert scale 1–7).

As can be seen in Figure 2, accuracy (indicating that a neuromyth was false) was higher after a month following both feedback conditions compared to no feedback. For a complete record of coefficients, see Table 5. Accuracy differed for the feedback-only and feedback–explanation conditions. Participants who received an explanation were 0.47 more likely to indicate that the neuromyths were false than participants in the feedback-only condition. These results suggest that providing an explanation may improve the effectiveness of feedback after a one-month delay compared to providing simple feedback. The findings from Experiment 2 align with previous research showing that adding explanations to refutations can increase accuracy in detecting misconceptions after a delay (Rich et al. 2017; Swire et al. 2017). However, the results deviate from Experiment 1, where there was no difference between the type of feedback on accuracy at the one-week delay.

As in Experiment 1, higher accuracy on the initial test predicted higher accuracy on the delayed test. Also, higher confidence in responses on the delayed test predicted higher accuracy on the delayed test. Higher initial confidence in beliefs predicted higher accuracy on the delayed test. However, deviating from Experiment 1, confidence in delayed test judgments and the influence of behavior were not significant predictors.

#### 2.2.3. Effect of the Corrective Feedback on Reasoning

As in Experiment 1, we also examined the effect of feedback on reasoning described in the scenarios. As can be seen in Table 6, receiving feedback (both feedback only and explanation–feedback) significantly increased reasoning accuracy (disagreement with reasoning based on neuromyths) compared to not receiving feedback. These findings replicate those from Experiment 1, suggesting that reasoning about neuromyths can be corrected using feedback. However, deviating from Experiment 1, which used a shorter delay, in Experiment 2, receiving an explanation, compared to simple feedback, significantly improved reasoning accuracy. Also, deviating from Experiment 1, confidence in initial beliefs and reliance on statements in life did not significantly predict reasoning accuracy.

## 3. Experiment 3

Results from Experiment 1 using a one-week delay suggested that both simple feedback and feedback with an explanation were effective for correcting erroneous beliefs and predicting more accurate reasoning. However, after a one-month delay (Experiment 2), receiving feedback that included an explanation was more effective for updating beliefs and predicting more accurate reasoning about real-world learning scenarios. In Experiment 3, we investigated whether these results would replicate in a sample of educators. To date, no study has investigated if feedback can be used to correct teachers’ *reasoning* about learning practices. A recent study by Ferrero et al. (2020) found that refutations can be used to update educators’ knowledge of neuromyths; however, refutations did not change teachers’ intention to implement the neuromyths in their teaching practices. This finding suggests that there is discrepancy between the efficiency of the refutations for updating beliefs compared to influencing reasoning about these learning concepts. Therefore, in Experiment 3 we examined the effectiveness of different types of refutative feedback on teacher’s beliefs and reasoning using neuromyths. We examined the effect of refutative feedback on beliefs and reasoning after a delay using similar procedures to those used in Experiments 1 and 2.

### 3.1. Method

#### 3.1.1. Participants

Based on previous work on correcting neuromyths using a similar design (Ferrero et al. 2020), we selected an effect size of *t* = 5.72, 1– β = 0.80 power at α = 0.05 to calculate the number of participants that would be needed to detect a significant difference between each of the feedback conditions using a 75% assurance to control for uncertainty. This power analysis suggested that we needed a sample size of 54 participants (18 participants per condition). As in Experiments 1 and 2, we recruited additional participants to ensure that we would obtain complete data for at least 54 participants. Because Experiment 3 was conducted across multiple sessions and thus attrition would be an issue with this study design (Keith et al. 2017), we recruited a fairly high number of participants.

Four hundred and one educators were recruited using Amazon’s Mechanical Turk (Mturk). To ensure recruitment of our demographic of interest, only those who had “employment industry—education” listed as their occupation category were able to see the human intelligence task (HIT) on Mturk. Potential participants then completed two yes/no qualification questions (“Are you currently employed as a teacher” and “Do you currently teach to more than ten people on a weekly basis?”). If participants answered “yes” to both questions, they could accept the HIT. To promote data quality, participants were required to have completed at least 50 HITS and have at least a 90% approval rate based on these previously conducted HITs. The final data set contained 104 participants, 23–69 years old, *M* age = 38.26 *SD* = 12.37). Participants received $3.00 to complete all parts of the study. Despite attempts to encourage teachers to return after the one-month delay (i.e., multiple reminder emails), Experiment 3 had a large amount of attrition, resulting in a very small sample at the one-month test (*n*= 51), so the one-month results are not reported in-text. Out of the 104 participants included, 37 participants responded to the descriptive questions. The low response rate on the descriptive questions is partly due to the fact that descriptive questions were placed in the one-month-delayed survey. Out of the participants answering the descriptive questions, 57% of the teachers had a bachelor’s degree, 37% had a master’s degree, and 3% had other academic degrees. Participants taught at the following levels: 3% preschool, 5% primary school (ages 3–5), 30% elementary (ages 4–11, grades K–5), 24% middle school (ages 11–14, grades 6–8), 19% high school (ages 14–18, grades 9–12), and 19% higher education/postsecondary (college or university). On average, teachers had 13 years of teaching experience (*M* = 13.42, *SD* = 11.10). The educational subjects teachers mainly taught were as follows: 41% math and science, 16% social science, 17% computer technology, 4% fine arts, language 14%, and 8% other subjects.

#### 3.1.2. Design and Materials

The design and materials were identical to those used in Experiments 1 and 2, with two exceptions. Some scenarios were adapted to better align with an educator’s perspective of the neuromyths. Also, to better align with the sample of interest, the question assessing influence on daily life was changed to *“This concept has influenced my teaching practices”*.

#### 3.1.3. Procedure

The procedure was identical to that used in Experiments 1 and 2 but with some minor changes. Instead of only returning one time after the initial test, participants returned twice—after a one-week and one-month delay. Participants enrolled in the study by accepting a HIT on MTurk. After accepting the HIT, participants were directed to the study administered in Qualtrics.

#### 3.1.4. Analytic Plan

The analysis plan was identical to that used in Experiment 2.

### 3.2. Results and Discussion

#### 3.2.1. Initial Beliefs

The results showed that teachers performed worse than chance on the initial test (*M* = 0.30, *t*(123) = −13.39, *p* = < .01, *d* = 1.32). Teachers had lower accuracy than students in Experiments 1 and 2. As can be seen in Table 7, teachers in the feedback-only condition had lower initial mean accuracy than participants in the other conditions; however, this difference could be explained by chance, *F*(2, 101) = 1.83, *p* = 0.17). As in Experiments 1 and 2, we also controlled for initial accuracy in the following analysis.

#### 3.2.2. Effect of Feedback on Statement Accuracy

Both feedback conditions significantly improved participants’ accuracy after a one-week delay compared to receiving no feedback. For a complete record of coefficients, see Table 8. As in Experiment 1—which also used a one-week delay—both the feedback-only and feedback–explanation conditions similarly increased accuracy. In addition, the more teachers indicated that a neuromyth had influenced their teaching practice, the less likely they were to correct it on the delayed test overall. However, neither initial confidence nor delayed test confidence significantly increased accuracy on the delayed test.

#### 3.2.3. Effect of Corrective Feedback on Reasoning Accuracy

**Figure 3:**
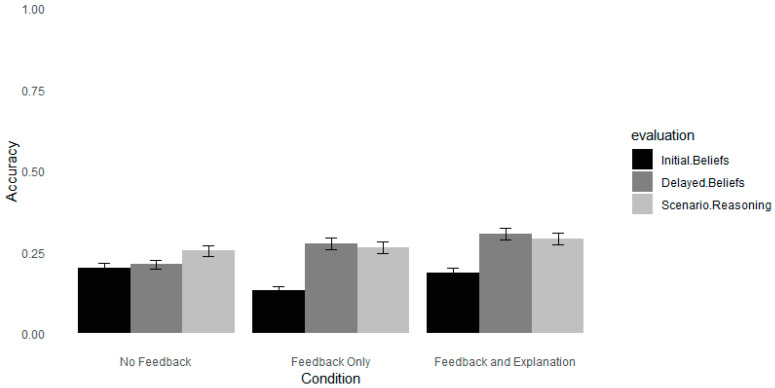
The accuracy of evaluating neuromyths and scenarios in Experiment 3, one-week delay. *Note*. Error bars depict standard deviations. Accuracy was calculated as the average score recorded on the Likert scale (Likert scale 1–7).

As in the previous experiments, we investigated whether feedback influenced participants’ ratings of the reasoning illustrated in the scenarios. As shown in Table 9, the accuracy of teachers’ ratings were not significantly improved following either type of feedback compared to no feedback (see Figure 3). On the one-week test, higher confidence predicted higher accuracy in ratings of reasoning. In addition, the more teachers indicated that a neuromyth had influenced their teaching practice, the less accurate they were on the delayed test.

## 4. General Discussion

Research suggests that students and educators often endorse neuromyths—unsubstantiated beliefs about learning and intelligence (Dekker et al. 2012; Lithander et al. 2021). Ascribing to these false beliefs can lead students to choose suboptimal study strategies and educators to teach using these educational practices (Dunlosky et al. 2013; Morehead et al. 2016). Therefore, we investigated whether feedback on the accuracy of neuromyths would influence students’ and teachers’ reasoning about learning. The results showed that both students and teachers hold false beliefs about learning and the brain, in line with previous findings (Dekker et al. 2012; Ferrero et al. 2016; Howard-Jones 2014; Lithander et al. 2021). Initial beliefs in neuromyths were high across all three experiments. For example, between 79% and 94% of participants stated that they believed individuals learn better when they receive information in their preferred learning style.

However, the effectiveness of feedback on beliefs differed across the experiments, suggesting that the effectiveness of feedback may be influenced by the test delay and the population. In Experiment 1, students who received either type of feedback (feedback only or feedback and explanation) had significantly higher accuracy than those who received no feedback on the delayed test. There was no significant difference in accuracy between the feedback-only and feedback–explanation conditions, replicating Lithander et al. (2021). In contrast, in Experiment 2, students who received feedback with an explanation had significantly higher accuracy on the delayed test relative to students who received feedback only. However, participants in both feedback conditions had higher accuracy than those in the no-feedback condition. This finding diverges from the results of Lithander et al. (2021). Still, it aligns with frameworks suggesting that explanations can help people better integrate the correction into long-term memory (Kendeou and O’Brien 2014) and research showing the benefits of providing explanations (Rich et al. 2017; Swire et al. 2017). The differing effects of explanations on accuracy between Experiments 1 and 2 may be due to the difference in delay (one week versus one month). Providing simple feedback may be sufficient to correct neuromyths at a short delay, whereas explanations may be needed to correct neuromyths after a longer delay. Diverging from the students’ results, teachers (Experiment 3) who received feedback only were not significantly more accurate on the one-week delayed test than teachers who received no feedback. However, receiving feedback and an explanation significantly improved accuracy for teachers on the one-week delayed test. This finding may suggest that teachers (relative to students) are more resistant to updating neuromyths and may need an explanation to change their beliefs.

As described above, feedback influenced later belief accuracy. In addition to the influence of feedback on accuracy, some other predictors contributed to whether beliefs were updated. Across all three experiments, higher initial accuracy predicted accuracy on the delayed test. In Experiment 1, greater confidence in beliefs on the delayed test predicted more accurate responding to the statements. Students who reported higher reliance on neuromyths in their daily lives were less likely to update their beliefs. In both Experiments 1 and 2, students who were more confident in their beliefs were more likely to be accurate. In contrast to Experiment 1, in Experiment 2 confidence on the initial test increased the likelihood of belief updating. One potential reason for this discrepancy (despite the same stimuli and similar participants) is the difference in delay. In Experiment 3, feedback condition and reliance on neuromyth were the only significant predictors of belief updating for teachers. As with students in Experiment 1, higher reliance on neuromyths decreased the probability of accuracy on the delayed test.

In addition to examining change in beliefs accuracy, another main goal was to investigate students’ and teachers’ reasoning using neuromyths following feedback. For students, the effects of feedback on reasoning closely mimicked the effects obtained for beliefs (described above). In Experiment 1 (after a one-week delay), students in the feedback-only or feedback-and-explanation conditions showed better reasoning than students who received no feedback. In Experiment 2, students who received feedback with an explanation showed better reasoning than students who received feedback only or no feedback. However, receiving feedback only still predicted more accurate reasoning than receiving no feedback. Once again, this difference in student performance may relate to the length of the delay between the correction and test (one week vs. one month). Unlike students whose reasoning may be influenced by a neuromyth correction, the teacher’s reasoning was unaffected by feedback. After a one-week delay, there was no difference in reasoning accuracy across the three conditions. This finding suggests that teachers may continue to rely on neuromyths when reasoning about real-world learning situations. This finding also aligns with the literature indicating that people may continue to rely on erroneous information when reasoning about the world, even though they are aware that the information has been corrected (Guillory and Geraci 2016; Lewandowsky et al. 2012; Walter and Tukachinsky 2020). It is noteworthy that, across all three experiments, initial accuracy in neuromyth evaluation (correctly identifying neuromyth) significantly improved reasoning accuracy on the delayed test. Similarly, across all experiments, higher confidence in reasoning was associated with a higher accuracy of reasoning. In addition to these predictors, higher reported reliance on neuromyths reduced teachers’ (but not students’) accurate reasoning. 

One practical implication of these results is that feedback should be designed to target erroneous reasoning and not just erroneous beliefs. Rather than simply correcting erroneous statements (“people have different learning styles”), it may be key to correct the reasoning and behaviors that result from reliance on neuromyths (that material should be presented in different formats for different students; that one should listen to an audio book to remember it if deemed an auditory learner). Because these neuromyth-based practices may be well engrained and may operate on a more implicit level (see Rousseau 2021, for review), it may be necessary for people to practice applying this newly-updated knowledge in various real-world situations to overcome automatic tendencies to rely on neuromyths to make study and teaching decisions. Of course, it is also possible that though people know that the neuromyth is wrong, they continue to hope and believe that it might be effective (see Guillory and Geraci 2010). If this is the case, other interventions that target this type of motivational reasoning may be needed.

Specifically, these types of interventions may be important for teachers who seem, based on the current data, less likely to apply corrections of neuromyths to their reasoning. Teachers may be more resistant to corrections relative to students given that they may have known and relied on neuromyths for longer. Furthermore, over the course of teachers’ careers, they may be more likely to be exposed to misconceptions (neuromyths) in workshops, conferences, and educational materials that promote neuromyths for classroom use (Busso and Pollack 2015; Goswami 2006). Future studies might examine this hypothesis. Also, future studies might focus on increasing the source’s trustworthiness to attenuate the influence of misconceptions about learning and the brain on reasoning. Though we included citations to scientific sources in the feedback-and-explanation condition, this design feature may not be sufficient to help people update their knowledge, as other studies suggest that trustworthiness is more important than expertise for the correction of erroneous information (Ecker and Antonio 2021; Guillory and Geraci 2013).

Although our findings suggest that beliefs in neuromyths and reasoning about neuromyths can be corrected with feedback, there is little consensus about how changing beliefs might translate into teaching practices, and future research should examine this issue. Some suggest that beliefs in neuromyths do not influence educators’ teaching practices (Krammer et al. 2021). On the other hand, two recent studies indicated that beliefs in neuromyths do transfer to teaching practices (Blanchette Sarrasin et al. 2019; Hughes et al. 2020). In a large online questionnaire from Quebec, Blanchette Sarrasin et al. (2019) found that many participants (74%) endorsed the learning style myth. Out of those participants, a majority (64.9%) regularly implemented this practice in the classroom. Only a small minority (2.4%) of those who endorsed the learning style myth reported not implementing this practice in their classroom teaching. With these mixed results, future studies should include behavioral measures to investigate if neuromyths affect teaching practices and how correcting them may influence those practices. As others have discussed, this work should be conducted closely parallel to classroom practices (Rousseau 2021; Torrijos-Muelas et al. 2021). One technique that has been found to be effective in various formats, including classroom teaching, is using contrasting cases to provide feedback (Loibl and Rummel 2014). Future studies should explore contrasting students’ beliefs about educational misconceptions and using feedback that explicitly contrasts erroneous beliefs with the correct information. Such an approach has promoted long-term conceptual change (Asterhan and Dotan 2018). Contrasting feedback that addresses individual student errors and misconceptions could make the input more effective than generic explanatory refutation texts.

Future studies should also focus on creating effective interventions for students. We found that students often hold erroneous beliefs about how to learn, which is consistent with previous research (Blasiman et al. 2017; Dirkx et al. 2019; Morehead et al. 2016). Therefore, finding practical interventions that can help students use better study strategies is important. Recent studies have shown that it is possible to help students improve their study strategies by providing training programs that promote effective learning strategies and self-regulation (McDaniel and Einstein 2020). So far, such interventions have focused on promoting effective strategies. These interventions should also focus on correcting erroneous preconceptions about learning, as having erroneous preconceptions may impede future learning (Bransford et al. 2000; Gelman and Lucariello 2002; Lucariello et al. 2014). In other words, correcting erroneous beliefs and teaching students to use effective strategies are important for “making the truth stick and the myths fade” (Schwarz et al. 2016).

There are limitations to the current study. For example, in Experiment 1, we measured the endorsement of neuromyths using a standard true/false response scale. Recently, this scale has come under scrutiny because neuromyths may not encompass one coherent construct (Horvath et al. 2018; Krammer et al. 2021; Macdonald et al. 2017). This potential issue led us to conduct Experiments 2 and 3 using Likert scales, which have been adopted in several studies. However, future research is necessary to understand how best to measure beliefs in neuromyths. Also, we examined one sample of teachers, but the results may differ using a different sample of teachers. Future research should examine the effectiveness of corrections for various groups of teachers and attempt to tailor these corrections to these groups. Another potential limitation is that the data were collected online using MTurk. Of course, using an online sample can be a strength because it can allow for responses from a broad sample of teachers. However, some may worry about data quality from MTurk participants on the basis that these participants may not be paying attention and taking studies as seriously as other participants. To address this question, we examined participant’s performance on the math tests in sessions 1 and 2 as a proxy for attention check. The results showed that very few participants (1.35%) failed to answer all of the math problems within the allotted time (1.35% in part 1 and 1.45% in part 2), suggesting that we had very few cases of participants who were likely not attending to the task. On average, participants completed most of the math problems (76% in part 1 and 85.2% in part 2), and of these, they answered 86% and 88% of them correctly in sessions 1 and 2, respectively. The math problems had some complexity to them (e.g., (72 − 50) × 2 = ?; 10 + 7 − 5 = ?; 44 − 20 + 10 = ?), and participants were given only 6 s to complete each one, suggesting that participants needed to actively pay attention to this task to be able to make their responses within the tight time limit. Thus, the fact that most people completed the task on time and answered a relatively high percentage of the items correctly suggests that the majority of participants in Experiment 3 were paying attention to the study. In addition, the only participants included in the analyses were those who completed each session of the study, suggesting a relatively high degree of intention to take the study seriously.

In sum, the results of the current studies suggest that students and teachers can update initial erroneous beliefs about learning and the brain, partially replicating previous research. It is promising that simple feedback can help students to update erroneous beliefs and influence the reasoning because this feedback can be easily implemented in the classroom. However, more research is needed to understand how to make feedback effective so that educators do not rely on corrected beliefs when reasoning about learning. Based on the results of the current studies, educators may update their beliefs when given feedback. However, these updated beliefs do not translate into better reasoning about learning and the brain, where they may still rely on their original erroneous beliefs. For now, our results suggest that students’ and educators’ erroneous beliefs can be updated, but reasoning may not be influenced by this belief updating.

## Figures and Tables

**Table 1:** Correct responses to neuromyths for each feedback condition in Experiment 1 after initial test and a one-week delay.

Condition	Number of Participants (*n*)	Initial Test		Statements		Scenarios	
		*M*	*SD*	*M*	*SD*	*M*	*SD*
No Feedback	33	0.40	0.14	0.35	0.20	0.43	0.29
Feedback Only	34	0.41	0.16	0.73	0.18	0.66	0.32
Feedback and Explanation	43	0.41	0.13	0.74	0.24	0.68	0.30
	110	0.41	0.13	0.62	0.26	0.60	0.32

*Note.* Average and standard deviation values for scenarios are reported based on the Likert scales rescored to 0–1. Responses were considered correct if participants indicated any degree of disagreement (5, 6, or 7 on the Likert scale) with the faulty reasoning described in the scenarios or false statements.

**Table 2:** Estimates for delayed test belief (statements) in Experiment 1.

	Outcome				
*Predictors*	*Estimates*	*Odds Ratio*	*SE*	*p*	*z-Value*
Intercept	−1.22	0.30	0.27	<0.001	−4.54
Feedback-only (vs. control)	2.13	8.40	0.30	<0.001	7.17
Feedback–explanation (vs. control)	2.34	10.34	0.29	<0.001	8.17
Feedback–explanation (vs. feedback-only)	0.21	1.23	0.28	0.46	0.74
Initial accuracy (statement)	1.27	3.57	0.08	<0.001	8.71
Initial confidence in statement evaluation	0.06	1.07	0.76	0.45	0.76
Delayed confidence in statement evaluation	0.38	1.46	5.47	<0.001	5.47
Influenced behavior in life	−0.28	0.76	−3.22	<0.01	−3.22
**Random Effects**					
σ^2^		3.29			
τ_00ID_		1.08			
τ_00Question_		0.50			
ICC		0.51			
N_Question_		20			
N_ID_		110			
Total number of observations		2200			

*Note*. Conditional R^2^ = 0.515.

**Table 3:** Estimates for scenario reasoning in Experiment 1.

	Outcome			
*Predictors*	*Estimates*	*SE*	*p*	*df*
Intercept	3.45	0.20	<0.001	84.39
Feedback-only (vs. control)	1.25	0.22	<0.001	105.80
Feedback–explanation (vs. control)	1.39	0.20	<0.001	105.53
Feedback–explanation (vs. feedback-only)	0.14	0.20	0.48	105.07
Initial accuracy (statement)	0.50	0.08	<0.001	2089.28
Initial confidence in statement evaluation	0.01	0.04	0.77	2179.87
Delayed confidence in scenario evaluation	0.33	0.04	<0.001	2109.66
Influenced behavior in life	−0.03	0.05	0.46	2163.09
**Random Effects**				
σ^2^	3.01			
τ_00ID_	0.68			
τ_00Question_	0.29			
ICC	0.32			
N_Question_	20			
N_ID_	110			
Total number of observations	2200			

*Note*. Conditional R^2^ = 0.440.

**Table 4:** Correct responses to neuromyths for each feedback condition in Experiment 2 after a one-month delay.

Condition	Number of Participants (*n*)	Initial Test		Statements		Scenarios	
		*M*	*SD*	*M*	*SD*	*M*	*SD*
No Feedback	44	0.45	0.32	0.42	0.29	0.30	0.20
Feedback Only	38	0.42	0.31	0.54	0.31	0.36	0.21
Feedback and Explanation	40	0.45	0.32	0.63	0.30	0.41	0.21
	122	0.44	0.32	0.53	0.32	0.35	0.21

*Note.* Average and standard deviation values for scenarios are reported based on the Likert scales rescored to 0–1. Responses were considered correct if participants indicated any degree of disagreement (4 or 5 on the Likert scale) with the faulty reasoning described in the scenarios or false statements.

**Table 5:** Estimates for delayed test belief (statements) in Experiment 2.

	Outcome			
*Predictors*	*Estimates*	*SE*	*p*	*df*
Intercept	2.25	0.18	<0.001	103.49
Feedback-only (vs. control)	0.81	0.18	<0.001	118.08
Feedback–explanation (vs. control)	1.28	0.17	<0.001	118.06
Feedback–explanation (vs. feedback-only)	0.47	0.18	0.01	117.68
Initial belief (statement)	0.47	0.03	<0.001	2256.15
Initial confidence in statement evaluation	0.09	0.04	0.02	2430.29
Delayed confidence in statement evaluation	0.08	0.04	0.03	2422.69
Influenced behavior in life	−0.02	0.04	0.67	2298.90
**Random Effects**				
σ^2^	1.92			
τ_00ID_	0.21			
τ_00Question_	0.53			
ICC	0.28			
N_Question_	20			
N_ID_	122			
Total number of observations	2440			

*Note.* Conditional R^2^ = 0.418.

**Table 6:** Estimates for reasoning about scenarios in Experiment 2.

	Outcome			
*Predictors*	*Estimates*	*SE*	*p*	*df*
Intercept	2.18	0.13	<0.001	95.86
Feedback-only (vs. control)	0.40	0.13	<0.001	117.87
Feedback–explanation (vs. control)	0.65	0.13	<0.01	117.79
Feedback–explanation (vs. feedback-only)	0.25	0.13	<0.01	117.42
Initial belief (statement)	0.22	0.02	<0.001	2331.56
Initial confidence in statement evaluation	−0.01	0.03	0.79	2429.90
Delayed confidence in scenario evaluation	0.11	0.02	<0.001	2417.42
Influenced behavior in life	−0.04	0.03	0.22	2352.60
**Random Effects**				
σ^2^	0.93			
τ_00ID_	0.30			
τ_00Question_	0.13			
ICC	0.32			
N_Question_	20			
N_ID_	122			
Total number of observations	2440			

*Note.* Conditional R^2^ = 0.396.

**Table 7:** Accuracy in correctly indicating that a false statement is false for each feedback condition in Experiment 3 with teachers tested after a one-week and a one-month delay.

Condition	Number of Participants (*n*)	Initial Test		One-Week Delay Statements		One-Week Delay Scenarios	
		*M*	*SD*	*M*	*SD*	*M*	*SD*
No Feedback	38	0.32	0.30	0.33	0.30	0.35	0.31
Feedback Only	31	0.26	0.27	0.35	0.36	0.35	0.35
Feedback and Explanation	35	0.32	0.30	0.41	0.36	0.41	0.35
	104	0.30	0.29	0.36	0.34	0.37	0.34

*Note.* Average and standard deviation values for scenarios are reported based on the Likert scales rescored to 0–1. Responses were considered correct if participants indicated any degree of disagreement (4 or 5 on the Likert scale) with the faulty reasoning described in the scenarios or false statements.

**Table 8:** Estimates for delayed test belief (statements) in Experiment 3 for one-week delay.

	Outcome			
*Predictors*	*Estimates*	*SE*	*p*	*df*
Intercept	1.62	0.14	<0.001	136.78
Feedback-only (vs. control)	0.24	0.18	0.19	95.12
Feedback–explanation (vs. control)	0.36	0.17	0.04	94.43
Feedback–explanation (vs. feedback-only)	0.12	0.18	0.51	95.32
Initial belief (statement)	0.29	0.02	<0.001	1988.39
Initial confidence in statement evaluation	−0.03	0.03	0.29	2038.18
Delayed confidence in statement evaluation	0.05	0.03	0.07	2057.33
Influenced behavior in life	−0.14	0.04	<0.001	2028.22
**Random Effects**				
σ^2^	0.89			
τ_00ID_	0.51			
τ_00Question_	0.02			
ICC	0.47			
N_Question_	20			
N_ID_	104			
Total number of observations	2067			

*Note.* Conditional R^2^ = 0.453.

**Table 9:** Estimates for reasoning about scenarios in Experiment 3 for one-week delay.

	Outcome			
*Predictors*	*Estimates*	*SE*	*p*	*df*
Intercept	2.06	0.15	<0.001	139.68
Feedback-only (vs. control)	0.08	0.19	0.87	95.13
Feedback–explanation (vs. control)	0.25	0.19	0.21	94.59
Feedback–explanation (vs. feedback-only)	0.17	0.20	0.39	95.21
Initial belief (statement)	0.14	0.02	<0.001	2005.41
Initial confidence in statement evaluation	−0.00	0.03	0.89	2034.58
Delayed confidence in scenario evaluation.	0.06	0.03	0.03	2049.08
Influenced behavior in life	−0.16	0.04	<0.001	2043.32
**Random Effects**				
σ^2^	0.98			
τ_00ID_	0.59			
τ_00Question_	0.04			
ICC	0.39			
N_Question_	20			
N_ID_	104			
Total number of observations	2067			

*Note*. Conditional R^2^ = 0.421.

## Data Availability

Data is available on OSF: https://osf.io/s85e4/?view_only=861260760bdc412a8d48e8eb8f5083b1 (accessed on 11 September 2024).
